# First-line treatment for advanced ovarian cancer: paclitaxel, platinum and the evidence

**DOI:** 10.1038/sj.bjc.6600567

**Published:** 2002-10-07

**Authors:** J Sandercock, M K B Parmar, V Torri, W Qian

**Affiliations:** Department of Public Health and Epidemiology, University of Birmingham, Birmingham, UK; MRC Clinical Trials Unit, London, UK; Istituto de Richerche, Farmacologiche ‘Mario Negri’, Milano, Italy

**Keywords:** ovarian cancer, platinum, paclitaxel, heterogeneity

## Abstract

Four large randomised trials of paclitaxel in combination with platinum against a platinum-based control treatment have now been published in full, representing around 88% (3588 out of 4057) of patients randomised into the eight known trials of this question. There is substantial heterogeneity in the results of these four trials. Four main explanations for this heterogeneity have been proposed: differences in the extent and timing of ‘crossover’ to taxanes in the control groups; differences in the types of patient included; differences in the effectiveness of the research regimens used; differences in the effectiveness of the control regimens used. In this study we examine whether any of these explanations is consistent with the pattern of results seen in these trials. Each explanation suggests that a particular characteristic of each trial was responsible for the results observed. For each explanation the trials were split into groups according to that characteristic, in order to partition the total heterogeneity into that seen ‘within’ and ‘between’ groups of trials. If a particular explanation was consistent with the pattern of results, we would expect to see relatively little heterogeneity within each group of trial results viewed in this way, with most of the heterogeneity being between groups which are dissimilar with respect to the key characteristic. Heterogeneity ‘within’ and ‘between’ groups was formally compared using the *F*-ratio. If any explanation appeared to be consistent with the results of the trials, it was considered whether the explanation was also consistent with other evidence available about these regimens. Only one explanation appeared to be consistent with the pattern of results seen in these trials, and that was differences in effectiveness of the control arms used in these trials. This suggests that the very positive results in favour of paclitaxel/cisplatin seen in two of the trials may have been due to the use of a suboptimal control arm. There is no direct evidence about the relative effectiveness of the control arms used in these trials, but indirect evidence is consistent with the conclusion that the cyclophosphamide/cisplatin regimen used in two of the trials may be less effective than the control regimens used in the other trials. Specific concerns about the choice of a cyclophosphamide/cisplatin control arm in the first of these trials to report were raised before the results of the other trials were known, i.e. before any heterogeneity had been observed. Further investigation of this question would be useful. In the meantime, given all of the randomised evidence on the efficacy and toxicity associated with the regimens used in these trials, we conclude that single agent carboplatin is a safe and effective first-line treatment for women with advanced ovarian cancer.

*British Journal of Cancer* (2002) **87**, 815–824. doi:10.1038/sj.bjc.6600567 www.bjcancer.com

© 2002 Cancer Research UK

## BACKGROUND

In many countries the combination of carboplatin plus paclitaxel has become a standard first-line therapy for women requiring chemotherapy for ovarian cancer. Recently, however, evidence has emerged questioning this approach. A systematic review by the NHS Centre for Reviews and Dissemination in York, commissioned by the NHS R&D Health Technology Assessment Programme (HTA) on behalf of the National Institute for Clinical Excellence (NICE), reviewed four trials that compared standard platinum-based treatment against the combination of platinum and paclitaxel (Lister-Sharp *et al*, 2000; Taxanes, 2002). At the time of this review three of the four trials remained unpublished. These trials have now all been published in full. In this study we examine the most recent published results of the trials identified by this review. Full details of the search strategy, methods and results of the original review are contained in the assessment report produced for NICE, which is published in monograph form by the HTA Programme (Lister-Sharp *et al*, 2000; Taxanes, 2002; National Institute of Clinical Excellence, URL http://www.nice.org.uk/).

Although there are a number of differences between these trials, they all posed the same general question (Table 1

**Table 1:**
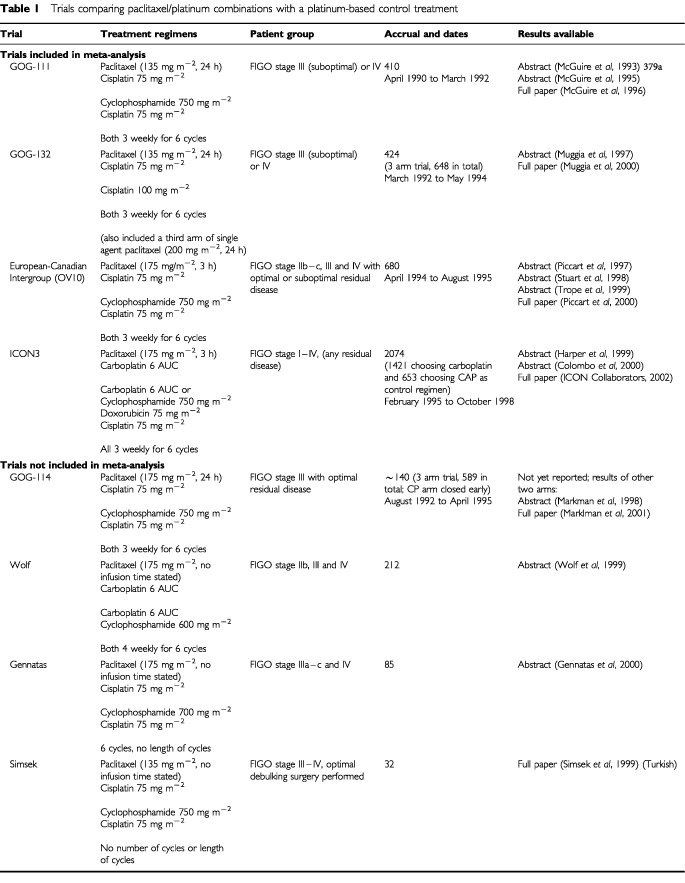
Trials comparing paclitaxel/platinum combinations with a platinum-based control treatment

). The first of these trials was GOG-111 (Mcguire *et al*, 1993, 1995, 1996), conducted by the Gynecology Oncology Group (GOG) in the United States. This was first presented at the annual meeting of the American Society of Clinical Oncology (ASCO) in 1993 and published in full in 1996. In GOG-111 410 patients were randomised and the trial reported very positive results in favour of paclitaxel/cisplatin compared with cyclophosphamide/cisplatin with hazard ratio of 0.61 (95%CI 0.47–0.79) for overall survival. These results were later confirmed by a European-Canadian Intergroup trial (OV10) (Piccart *et al*, 1997, 2000; Stuart *et al*, 1998; Trope and Vergote, 1999), which randomised 680 patients between the same two treatments and reported preliminary results at ASCO in 1997 and was published in May 2000. However, these results were contradicted by a further GOG trial, GOG-132 (Muggia *et al*, 1997, 2000), comparing single agent cisplatin against the same paclitaxel/cisplatin combination used in GOG-111, with 424 patients randomised between these two arms (648 randomised in total between three arms, with the third arm being single agent paclitaxel). The results of GOG-132, also presented at ASCO in 1997 and published in January 2000, were very different, suggesting no benefit to the paclitaxel/cisplatin regimen.

These data have generated considerable controversy. Although several possible explanations were suggested at the time, it was difficult to draw firm conclusions as to the appropriate interpretation of the conflicting results (Muggia *et al*, 1997, 2000; Sandercock *et al*, 1998). However, we now have the results of a fourth trial, ICON3 (Harper, 1999; Colombo, 2000; ICON Collaborators, 2002), which might help to clarify some of these issues. ICON3 compared paclitaxel/carboplatin against a control arm of single-agent carboplatin or the three drug CAP combination (cyclophosphamide, doxorubicin and cisplatin). ICON3 closed to accrual of new patients in 1998 with 2074 patients randomised. The preliminary results were presented at ASCO in May 1999 and are now published in full by the International Collaborative Ovarian Neoplasm Collaborators (ICON Collaborators, 2002).

## THE EXPLANATIONS FOR THE RESULTS OF GOG-132

**Figure 1:**
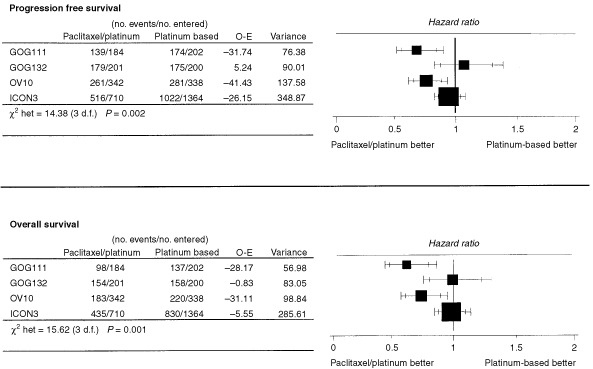
Results of trials comparing paclitaxel/platinum *vs* a platinum-based control arm.

The results of GOG-132 were somewhat unexpected, given the earlier results of GOG-111 and the preliminary data from OV10. The GOG-132 investigators put forward a number of possible explanations for their unexpected results including the play of chance, the different control regimens used in the trials, and the fact that a high proportion of patients on the single agent cisplatin (control) arm in GOG-132 went on to receive some treatment with paclitaxel prior to clinical evidence of disease progression. In relation to this last point, it was reported that around half of the patients on each of the three arms of the trial had gone on to receive some form of additional treatment after protocol chemotherapy but before progression of disease, with a proportion of those on the single agent cisplatin control arm receiving paclitaxel at this time. This early crossover has been widely assumed to account for the failure to detect any benefit for paclitaxel/cisplatin in the trial (Gore *et al*, 1997; Adams *et al*, 1998; National Cancer Guidance Steering Group, 1999). However, a number of individuals, including the GOG-132 investigators (Muggia *et al*, 2000; Torri *et al*, 2000), have been reluctant to accept that this explanation is necessarily sufficient to explain the difference in the results. The most obvious difficulty is that only a minority (24%) of the single agent cisplatin control group crossed over to paclitaxel before progression. Whilst this is certainly a substantial minority, if the true difference between single agent cisplatin as used in GOG-132 and paclitaxel/cisplatin were as large as the difference observed in GOG-111 and OV10 (both using a control arm of cyclophosphamide/cisplatin) it seems unlikely that crossover to paclitaxel in just one quarter of the control patients could apparently eliminate the difference between the two regimens, i.e. to give a relative risk of death (hazard ratio) of around one for both progression-free survival and overall survival (see Figure 1

).

The credibility of the notion that crossover could be solely responsible for the unexpected results of GOG-132 is further questioned if comparisons between response rates are examined. Although not as reliable or perhaps clinically relevant as the endpoints of progression-free and overall survival, this is the only outcome measure which could not have been affected by early crossover in any of the trials. Both GOG-111 and OV10 report large and statistically significant differences in clinical response rates in favour of paclitaxel/cisplatin; overall (complete plus partial) response rate 60% *vs* 73% (*P*=0.01) in GOG-111 and 45% *vs* 59% (*P*=0.01) in OV10. In contrast, GOG-132 reports identical response rates for single agent cisplatin as for cisplatin/paclitaxel, 67% *vs* 67% respectively, with complete responses in 42% *vs* 43%, respectively. Thus the inconsistency in the results of GOG-132 as compared with GOG-111 and OV-10 is present in endpoints measured both before and after any crossover occurred, giving further reason to doubt the adequacy of ‘early crossover’ as an explanation for the different results.

## RESOLVING SOME UNANSWERED QUESTIONS

The results of these four trials (GOG-111, GOG-132, OV10 and ICON3) have now all been published in full (Stuart *et al*, 1998; Muggia *et al*, 2000; Piccart *et al*, 2000; ICON Collaborators, 2002) and it therefore seems appropriate to reassess all the evidence. The total number of patients in these four trials is 3588. We have identified four further trials in Table 1 (Simsek *et al*, 1999; Wolf *et al*, 1999; Gennatas *et al*, 2000; Markman *et al*, 2001). The results of these trials are not available in any usable form for the following reasons. The GOG-114 trial (Markman *et al*, 2001) (with approximately 140 patients) as far as we are aware has never reported any results for the (non-taxane-containing) control arm, the trials of Wolf *et al* (1999) (212 patients) and Gennatas *et al* (2000) (89 patients) have been reported in abstract form only, while the small trial of Simsek *et al* (1999) (32 patients) has been reported in Turkish only and it has not been possible to extract relevant information. Thus this review is based on the four largest trials from which we can extract relevant data. These trials represent 88% (3588 out of 4057) of all the patients randomised into known trials of this question.

A naive meta-analysis, which simply pools results without paying attention to differences in the results of four trials, would suggest a benefit to paclitaxel/platinum, smaller than that originally expected on the basis of GOG-111, but still statistically (and perhaps clinically) significant. Using the ‘fixed effects’ model, the pooled result for progression-free survival gives an estimated hazard ratio of 0.87 with a 95% confidence interval of (0.80, 0.94), *P*=0.0003. The pooled result for overall survival is similar, with a pooled hazard ratio of 0.88, 95% confidence interval of (0.81, 0.96), *P*=0.004.

However, there is clear statistical heterogeneity in these results; for progression-free survival χ^2^_(het)_=14.37 (3 d.f.), *P*=0.002 and for overall survival χ^2^_(het)_=15.62 (3 d.f.), *P*=0.001. There is therefore strong evidence that these trials are not providing answers to the same question. Simply pooling the results under these circumstances is inappropriate. An alternative sometimes used in these circumstances is a ‘random effects’ model. Using this approach, the pooled results for progression-free and overall survival give estimated hazard ratios of 0.84 with a 95% confidence interval of (0.70, 1.01; *P*=0.06) and 0.82 (0.66, 1.02; *P*=0.08) respectively. However, the ‘random effects’ analysis is far from satisfactory, because it is very sensitive to the assumptions underlying the ‘random’ element. A modest change in these assumptions (which are untestable) leads to very different results. Thus both of these analyses are not satisfactory. Further they leave the most important question unanswered: why is there such a clear conflict (heterogeneity) in the results of these four trials?

Four possible explanations for this heterogeneity are proposed: (1) differences in extent of crossover (including crossover prior to progression); (2) differences in the type of patients included in each trial; (3) differences in the research arms and (4) differences in the control arms.

We considered each of these hypotheses in turn. For each one, the approach used was to consider whether each explanation is plausible (could it give rise to heterogeneity) and, if so, whether it is consistent with the observed data (i.e. does it explain the heterogeneity observed), and if so, whether it is also consistent with evidence external to these trials.

We used a ‘meta-regression’ approach (Thompson and Sharp, 1999) to examine consistency between each explanation and the observed data. Each explanation suggests that a particular characteristic of each trial was responsible for the results observed. For each explanation we therefore split the trials into groups according to that characteristic, allowing us to partition the total heterogeneity into that seen ‘within’ and ‘between’ groups of trials. If a particular explanation was consistent with the pattern of results, we would expect to see relatively little heterogeneity within each group of trial results viewed in this way, with most of the heterogeneity being between groups which are dissimilar with respect to the key characteristic. Heterogeneity ‘within’ and ‘between’ groups was formally compared using the *F*-ratio. If a substantial proportion of the total observed heterogeneity is successfully ‘explained’ by the analysis, then the *F*-ratio comparing ‘between group’ to ‘within group’ heterogeneity should be large; the observed value may be compared to the corresponding F distribution to establish whether it is larger than could reasonably be expected by chance. Note that this is a one-sided test of the hypothesis that heterogeneity between groups is greater than that within groups; the hypothesis, that within group heterogeneity is greater, is of no interest here, and has no meaningful interpretation.

If any explanation appeared to be consistent with the results of the trials, we went on to consider whether the explanation was also consistent with other evidence available about these regimens.

## EXTENT OF CROSSOVER

The majority of patients whose disease progresses after ‘first-line’ chemotherapy will go on to receive further courses of chemotherapy (‘second-line’ treatment). This ‘second-line’ regimen used may be similar or different from that used first-line, depending on the observed duration of response to the agent(s) used first-line, the suitability of alternative agents and the preferences of both clinician and patient. Also patients who have a poor response to the original first-line treatment may switch to an alternative treatment in the absence of disease progression. Occasionally patients may receive ‘consolidation’ treatment after first-line treatment is completed but before clinical progression is noted and this may include additional cycles of the first-line regimen, an alternative chemotherapy regimen, or non-chemotherapy treatment (such as radiotherapy or hormonal treatment).

GOG-132 is the only trial to report substantial crossover to taxanes in the control arm prior to progressive disease being reported; OV10 reported 4% and ICON3 3% of control patients receiving taxane-based treatment at this time. No published information on additional treatments given prior to progression is available for GOG-111. However, in personal communication with the GOG it has been reported that very few patients in GOG-111 would have received any taxane therapy before progression on the control arm because of the limited availability of taxanes at that time. All trials apart from GOG-111 report substantial crossover in the platinum-based control arm to second-line taxane-based treatment after progressive disease was noted; GOG-111 reports that there was little crossover to taxanes in the control group.

**Figure 2:**
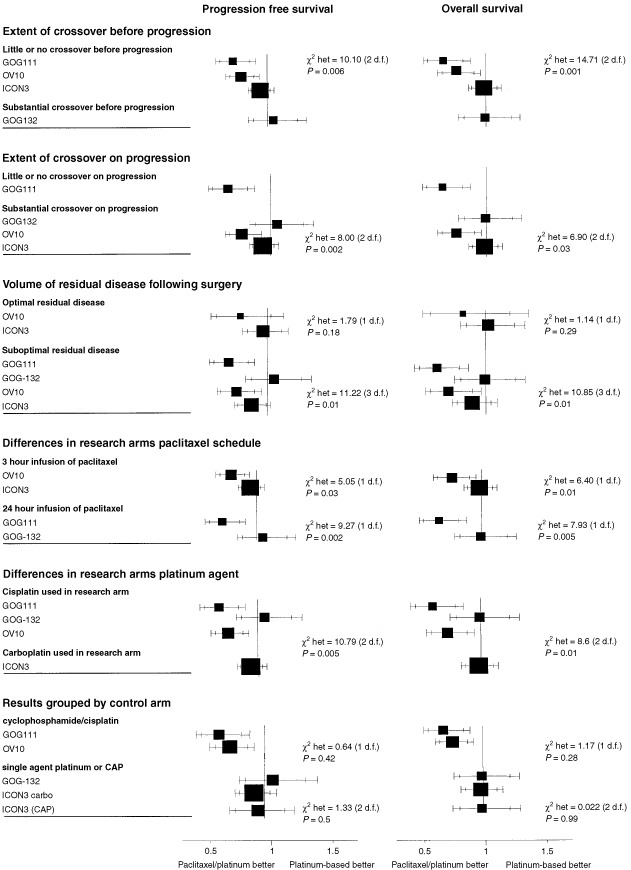
Results grouped by possible factors for heterogeneity.

Comparing groups with different amounts of crossover before progression (Figure 2

and Table 2

**Table 2:**
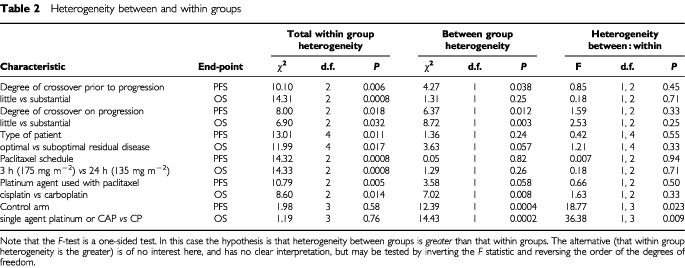
Heterogeneity between and within groups

) there is no indication that the heterogeneity between groups is substantially greater than that within groups for progression-free (*F*_1,2_=0.85, *P*=0.45) or overall survival (*F*_1,2_=0.18, *P*=0.71).

Within the group of trials reporting substantial crossover on progression heterogeneity between groups is not substantially greater than that within the groups (*F*_1,2_=2.53, *P*=0.25) for overall survival (Table 2). The results are similar for progression-free survival, although clearly the amount of crossover on progression could not reasonably be expected to influence these results.

This suggests that the heterogeneity in the results of these trials cannot be accounted for by crossover either before or after progression.

## TYPE OF PATIENT

It is possible for results of trials to differ because of differences in the types of patients involved, that is if certain treatments were more effective in some groups of patients than others. The types of patients included in each of the four trials are summarised in Table 3

**Table 3:**
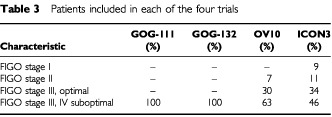
Patients included in each of the four trials

. Figure 2 shows the results of the trials grouped by the volume of residual disease remaining after surgery, with ‘suboptimal’ defined as >1 cm residual disease volume for GOG-111, GOG-132 and OV10, and >2 cm for ICON3; with the optimal groups being defined as the converse of these.

Comparing groups with optimally and suboptimally debulked disease, there is still substantial heterogeneity within groups. Heterogeneity between groups is not substantially greater than that within groups for progression-free (*F*_1,4_=0.42, *P*=0.55) or overall survival (*F*_1,4_=1.21, *P*=0.33).

This suggests that the heterogeneity in the results of these trials cannot be accounted for by differences in effectiveness for different types of patient.

## THE RESEARCH ARMS

Differences in the effectiveness of the research regimens used in the trials could account for the heterogeneous results observed. There are two differences in the paclitaxel/platinum regimens used in these trials. One is the scheduling of paclitaxel, 3 h infusion (with a dose of 175 mg m^−2^) or 24 h infusion (with a dose of 135 mg m^−2^), and the other is the platinum agent used, cisplatin or carboplatin.

Three trials (du Bois *et al*, 1999; Ozols *et al*, 1999; Neijt *et al*, 2000) have been conducted comparing cisplatin/paclitaxel against carboplatin/paclitaxel, one using different paclitaxel schedules on the two arms (see Table 4

**Table 4:**
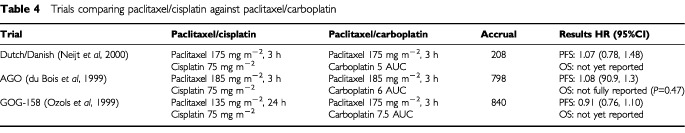
Trials comparing paclitaxel/cisplatin against paclitaxel/carboplatin

). Preliminary results of these trials do not suggest any difference in survival outcomes between the regimens compared; for this reason paclitaxel/carboplatin using a 3 h schedule of paclitaxel at 175 mg m^2^ is currently preferred on the basis of the more favourable toxicity profile of carboplatin and convenience of a shorter schedule (Markman *et al*, 1998; Neijt *et al*, 2000). However, this recommendation is based on preliminary data and more mature data should be available in the next year or so.

Looking within each group of results (Figure 2), by paclitaxel schedule and by platinum agent, there is still substantial heterogeneity within the groups. Comparing groups using different schedules of paclitaxel, heterogeneity between groups is not significantly greater than within groups; for progression-free survival (*F*_1,2_=0.01, *P*=0.94) or overall survival (*F*_1,2_=0.18, *P*=0.71) (see Table 2).

Comparing groups using different platinum agents in combination with paclitaxel, there is no evidence that heterogeneity between groups is substantially greater than within groups; for progression-free survival (*F*_1,2_=0.66, *P*=0.50) or overall survival (*F*_1,2_=1.63, *P*=0.33).

These results suggest that the heterogeneity in the results of these trials cannot be accounted for by differences in the research arms.

## THE CONTROL ARMS

The effectiveness of the control regimen, as well as that of the research regimen, is also of considerable importance in interpreting the results of any comparative trial.

Four different control regimens were used in these trials. GOG-111 and OV10 both used cyclophosphamide 750 mg m^−2^ combined with cisplatin 75 mg m^−2^; GOG-132 used single agent cisplatin 100 mg m^−2^; ICON3 allowed a choice (specified before randomisation) of carboplatin, dosed according to the area under the concentration-time curve (AUC) method (Calvert *et al*, 1989) at a minimum AUC of 6, or the combination of cyclophosphamide 500 mg m^−2^, doxorubicin 50 mg m^−2^ and cisplatin 50 mg m^−2^. The control regimens used in all trials were given 3 weekly for 6 cycles.

The two control regimens used in ICON3 have been shown to be equivalent in terms of effectiveness in the earlier ICON2 trial (ICON Collaborators, 1998). A number of trials comparing carboplatin and cisplatin have been conducted; the meta-analysis of these trials performed by the Advanced Ovarian Cancer Trialists Group (AOCTG) shows no evidence of any difference in effectiveness between these two platinum analogues (AOCTG, 1998). In particular, three trials compared the two as single agents at doses similar to those used in GOG-132 and ICON3, and the results indicate that these two regimens are similar in effectiveness (AOCTG, 1998). The two control arms of ICON3 (single agent carboplatin and CAP) and that of GOG-132 (single agent cisplatin) are thus known to be approximately equivalent to each other in terms of effectiveness. The systematic reviews and meta-analyses conducted by the AOCTG (AOCTG, 1998) and ourselves (Sandercock, 1998) did not reveal any trials comparing any of these three regimens against the cyclophosphamide/cisplatin regimen used in GOG-111 and OV10. Figure 2 therefore shows the trials grouped according to the control regimens with GOG-111 and OV10 forming one group using the control regimen of cyclophosphamide and cisplatin and the ICON3 and GOG-132 trials forming the other group.

For both progression-free and overall survival there is no evidence of heterogeneity between GOG-111 and OV10, (*P*=0.42 and *P*=0.28 respectively). There is also no evidence of heterogeneity between the results of the trials which used a control arm of either single agent carboplatin, cisplatin or CAP (*P*=0.51 for progression-free survival, *P*=0.99 for overall survival). There is, of course, still substantial heterogeneity overall, but this is largely between these two groups. The *F*-ratio for progression-free survival is 18.77 (1,3 d.f.), *P*=0.023 and for overall survival is 36.38 (1,3 d.f.), *P*=0.009, indicating that for both endpoints there is substantially more heterogeneity between groups than within groups and that this difference is unlikely to have arisen by chance.

This analysis indicates that one explanation which is consistent with the conflicting results is that the control regimen used in GOG-111 and OV10 was not as good as the control regimens used in ICON3 and GOG-132. The external evidence base for the control regimens differing in their effectiveness is examined below.

## EXTERNAL EVIDENCE-BASE FOR THE CONTROL-ARM EXPLANATION

Having found a plausible and consistent explanation for the heterogeneity in the results of these trials, it is important to investigate further to check that this explanation is also consistent with data from other trials. In the following discussion we will focus on randomised controlled trials (RCTs) identified through three systematic reviews: (1) the systematic review and meta-analyses conducted by the Advanced Ovarian Cancer Trialists Group based on individual patient data from 37 trials relating to the use of platinum (AOCTG, 1998); (2) the results of a literature search performed for a systematic review of platinum dose and dose intensity in ovarian cancer (Sandercock, 1998). The electronic search strategy (cut-off date June 1998) was designed to pick up all references to RCTs in ovarian cancer which involved a platinum agent, and (3) the Ovarian Cancer Meta-analysis project (Ovarian Cancer Meta-Analysis Project, 1991) based on individual patient data from four trials of cyclophosphamide/cisplatin (CP) *vs* cyclophosphamide, doxorubicin and cisplatin (CAP).

The two control regimens used in ICON3, which are denoted CAP(500,50,50) and carbo(6) using notation in brackets to represent the doses of individual drugs, have been shown in ICON2 to be closely equivalent (ICON Collaborators, 1998); the estimated hazard ratio for progression-free survival is 0.92 with 95% confidence interval (0.81–1.04) and for overall survival is 1.00 (0.86–1.16).

The control regimen in GOG-132, single agent cisplatin at 100 mg m^−2^, P(100), has not been compared directly with either of these two regimens, but there is substantial evidence from individual patient data meta-analysis that cisplatin and carboplatin are equivalent, both as single agents and in combination regimens (AOCTG, 1998). In particular, this meta-analysis included three trials which had compared single agent cisplatin at 100 mg m^−2^ against single agent carboplatin at 400 mg m^−2^ (Adams *et al*, 1989; Mangioni *et al*, 1989; Taylor *et al*, 1994). These trials individually and together indicated equivalence between these regimens; the pooled hazard ratio was 1.01 (95%CI 0.81, 1.26) with no evidence of heterogeneity (χ^2^_(het)_=0.02, *P*=0.99). Although it is not possible to translate carboplatin doses calculated on the basis of body surface area directly to doses calculated by the AUC method, the protocol dose in ICON3 equates to an average body surface area (BSA) dose of around 350–400 mg m^−2^. There is thus a substantial body of randomised evidence demonstrating that all three of the control regimens used in GOG-132 and ICON3 are similar in effectiveness.

The systematic reviews did not identify any randomised evidence comparing the cyclophosphamide (750 mg m^−2^) and cisplatin (75 mg m^−2^), CP(750,75), regimen used in GOG-111 and OV10 to any of the regimens used in either GOG-132 or ICON3 and therefore it is not possible to investigate its comparative efficacy. It would certainly be unethical to conduct any further trials using CP(750,75) as there is clear evidence that it is substantially less effective than paclitaxel/cisplatin. There is, however, some indirect evidence about this regimen.

Concern about the choice of CP(750,75) as a control arm in GOG-111 was first raised in 1996 (Parmar and Sandercock, 1996) when the results of this trial were first published, prior to any results being available from the other trials. This concern was based on the results of the Ovarian Cancer Meta-analysis Project (1991), which used updated individual patient data from trials comparing cyclophosphamide and cisplatin (CP) with cyclophosphamide, doxorubicin and cisplatin (CAP). The estimated hazard ratio for overall survival was 0.85 in favour of CAP (95% confidence interval around this estimate of 0.75–0.98).

However, three of the four trials included in this meta-analysis gave CP and CAP at intervals of 4 weeks; CP and CAP were given at 3 week intervals in GOG-111, OV10 and ICON3. Furthermore, the trials included in the meta-analysis used CP regimens with doses of 500–1000 mg m^−2^ of cyclophosphamide and 50–60 mg m^−2^ of cisplatin, whereas GOG-111 and OV10 used cyclophosphamide at 750 mg m^−2^ and cisplatin at 75 mg m^−2^. Thus this meta-analysis cannot confirm the size of any difference between CAP (500, 50, 50) and CP (750, 75), both given for 6 cycles at 3 week intervals.

Although CP (750, 75) became widely used in the late 1980s and early 1990s, the justification supporting it comes from a trial reported in 1989 and updated in 1996. The justification for this CP (750, 75) regimen appears to be based, at least in part, on the results of a Scottish Group trial comparing CP (750,50) with CP (750,100), both given for 6 cycles at 3 week intervals. This trial, with 159 patients, suggested a benefit to CP (750,100), with an estimated hazard ratio for overall survival of 0.68 (95% CI 0.46–0.99), but with considerably greater toxicity observed in this arm (Kaye *et al*, 1996). Although not tested in the trial CP (750,75) became widely used as a standard treatment in Europe and North America on the basis of a clinical compromise between possibly greater effectiveness but greater toxicity of the higher dose regimen (Kaye *et al*, 1996, Mcguire *et al*, 1996). In their discussion of the Scottish trial results, Kaye *et al* (1996) noted that key questions regarding the optimal dose of platinum remained unanswered, but that research questions were becoming increasingly focused on the taxanes. They expressed the belief that the dose of platinum would continue to be investigated within this new generation of trials.

The only published trials we have identified which have directly used the CP(750,75) regimen, have used it as the control regimen against experimental treatments: three of the trials discussed in this study, GOG-111, OV10 and GOG-114, comparing it with paclitaxel/cisplatin; an Italian trial investigating weekly cisplatin (Bolis *et al*, 1997), and a Dutch trial investigating a complex 4 drug regimen given 5 weekly (Neijt *et al*, 1987). One very small German trial included a similar CP regimen, comparing CP (700,70) given 4 weekly, CAP (700, 70, 70) given 4 weekly and P (100) given 2 weekly; this trial randomised only 60 patients, 20 in each arm, and so the results are relatively uninformative (Richter *et al*, 1990).

There is therefore no direct evidence concerning the effectiveness of cyclophosphamide 750 mg m^−2^ and cisplatin 75 mg m^−2^ compared with any of the control regimens used in GOG-132 and ICON3. However, the limited indirect evidence available from the OCMP meta-analysis (Ovarian Cancer Meta-Analysis Project, 1991), along with the results of ICON2 (ICON Collaborators, 1998) and the AOCTG meta-analysis (AOCTG, 1998), does support the hypothesis that the cyclophosphamide/cisplatin control regimen used in GOG-111 and OV10 may be less effective than the control regimens used in GOG-132 and ICON3.

The suggestion that single agent platinum (cisplatin or carboplatin) may be more effective than a platinum-based combination (cyclophosphamide/cisplatin) may appear to be at odds with some of the data from the AOCTG meta-analysis. This included a summary of all randomised trials comparing single agent platinum against platinum in combination.

The pooled results of all of these trials suggested a benefit to combination regimens (AOCTG, 1998). However, it is useful to examine that analysis in more detail, separating the trials into those which used platinum at the same dose in both the single agent and combination arms and those that increased the dose in the single agent arm. A summary of these trials split in this way is given in Table 5

**Table 5:**
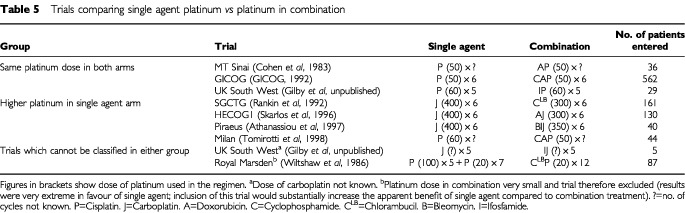
Trials comparing single agent platinum *vs* platinum in combination

and the meta-anaylsis plots are given in Figure 3

**Figure 3:**
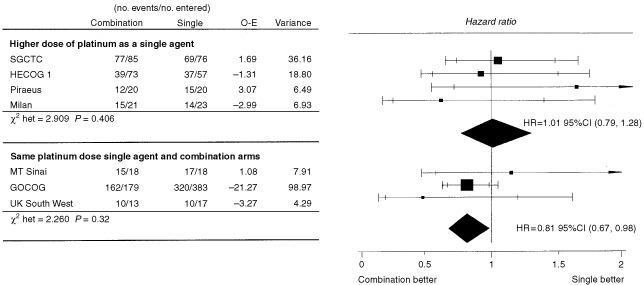
Randomised trials comparing platinum as a single agent with platinum in combination (Adapted from AOCTG, 1998).

The hazard ratio for overall survival when a higher platinum dose was used in the single agent arm was 1.01 (95% CI 0.79–1.28; *P*=0.96). This result of approximate equivalence is now also supported by ICON2 (hazard ratio of 1.00, 95% CI 0.86–1.16), and of course, GOG-132. We are grateful to a referee for pointing out a third trial published since the AOCTG update which randomised 176 women to receive either single agent cisplatin 75 mg m^−2^ or the combination of cyclophosphamide 500 mg m^−2^ and cisplatin 50 mg m^−2^. This trial (Marth *et al*, 1998) also supports the results shown in Figure 3, with a reported hazard ratio of 1.1 (95% CI 0.9, 1.3). It should be noted that the small Royal Marsden trial (Wiltshaw *et al*, 1986) would fall most naturally into this group of trials. Its extreme results in favour of the single agent would support and therefor enhance the argument made here, but we have not included it because it may be argued that a very low dose intensity of platinum was used in the combination arm.

In contrast when the platinum dose was not increased in the single agent compared to the combination arms, a hazard ratio for overall survival of 0.81 is observed (95% CI 0.67–0.98, *P*=0.03) in favour of combination treatment.

## DISCUSSION

Four hypotheses which have been proposed to explain the heterogeneous results of the four trials considered in this study have been examined. Only one of the proposed explanations appears to be consistent with the observed data. Differences in the effectiveness of the control regimens used is the only explanation proposed that can, in principle, account for the conflicting results of these trials.

It is possible that this is a chance finding and that the true, and as yet undetermined, explanation is different. It is worth noting, however, that whilst all of the explanations which have been examined could apply in theory to any set of trial results, in this case only one was proposed before any heterogeneity was observed; evidence suggesting that the control regimen used in GOG-111 was sub-optimal was pointed out before any heterogeneity had been observed (Parmar and Sandercock, 1996), that is before any results from OV10, GOG-132 or ICON3 were available.

It might be useful to further investigate these questions through meta-analysis based on individual patient data, and in particular to investigate the possible influence of patient characteristics on the results of these trials. In the meantime, the analyses and other evidence presented in this paper indicate the importance of giving platinum (either cisplatin or carboplatin) at optimal doses for the first-line treatment of women with advanced ovarian cancer. Given all of the randomised evidence on the effectiveness and toxicity of single agent cisplatin at 100 mg m^−2^, of the CAP (500, 50 and 50 mg m^−2^) combination and of all of the paclitaxel/platinum combinations, we conclude that optimal dose single agent carboplatin is a safe and effective first-line treatment for women with advanced ovarian cancer.
